# Brexpiprazole and Quetiapine Use During Pregnancy and Lactation: A Case Report

**DOI:** 10.1002/ccr3.73092

**Published:** 2026-07-06

**Authors:** Awateif Alsupiany

**Affiliations:** ^1^ Section of Women's Mental Health, Department of Mental Health National Guard Health Affairs Riyadh Saudi Arabia

## Abstract

Brexpiprazole, used off‐label in combination with quetiapine, may be a treatment option during pregnancy and breastfeeding when the risk of maternal relapse outweighs potential fetal or infant risks. This case describes perinatal use of polypharmacy without apparent adverse maternal or infant outcomes, underscoring the need for individualized, risk–benefit–based decisions in perinatal psychiatry. Findings should be interpreted cautiously given the absence of standardized assessments and the concomitant use of quetiapine.

## 1 Introduction

Brexpiprazole is a second‐generation (atypical) antipsychotic medication with partial agonist activity at dopamine D_2_ and serotonin 5‐HT_1_A receptors and antagonist activity at serotonin 5‐HT_2_A receptors [1]. It has been approved by the US Food and Drug Administration (FDA) for the treatment of schizophrenia and as adjunctive therapy for major depressive disorder [2]. Notably, brexpiprazole is not approved for the treatment of bipolar disorder; its use in this context is considered off‐label [2].

In the absence of randomized controlled trials guiding the management of bipolar II disorder, treatment recommendations are largely consensus‐based and aligned with those for bipolar I disorder. The Canadian Network for Mood and Anxiety Treatments (CANMAT) and the World Federation of Societies of Biological Psychiatry (WFSBP) provide guidance specific to bipolar II disorder and hypomania [3, 4, 5]. The National Institute for Health and Care Excellence (NICE) guidelines recommend antipsychotic treatment for mania or hypomania while considering patient preference, prior response, comorbidities, and adverse effects [6].

Use of antipsychotic medications during pregnancy has increased substantially [7], driven in part by increased fertility rates among women with mental illness [8] and expanding indications for atypical antipsychotics [9]. Discontinuation of treatment during pregnancy is associated with a significantly increased risk of relapse, potentially jeopardizing maternal and neonatal outcomes [10]. Data on brexpiprazole exposure during pregnancy and lactation remain extremely limited. A recent pharmacokinetic study by Konishi et al. [11] demonstrated placental transfer of brexpiprazole and reported low infant exposure during breastfeeding, providing the first quantitative data on this topic. That study involved a postpartum woman receiving brexpiprazole, quetiapine, risperidone, and its metabolite paliperidone, and measured drug concentrations in maternal plasma, cord blood, and breast milk. Brexpiprazole was detected in cord blood and breast milk at low levels, suggesting limited fetal and neonatal exposure. The present case adds clinically relevant observational data to this emerging body of evidence.

## 2 Case History/Examination

A 32‐year‐old woman with a longstanding history of bipolar II disorder and generalized anxiety disorder was referred to a specialized reproductive mental health service at approximately 32 weeks' gestation for medication management.

Her psychiatric history included recurrent hypomanic episodes characterized by decreased need for sleep, increased energy, impulsivity, and irritability, as well as recurrent depressive episodes marked by low mood, anhedonia, hypersomnia, and fatigue. She had no history of psychosis or suicide attempts and no significant medical comorbidities.

Regarding medication history and timeline: in August 2023, following a hypomanic episode, the patient was initiated on brexpiprazole 0.5 mg daily (subsequently titrated to 1 mg daily) and quetiapine 50–200 mg nightly, adjusted based on clinical response. In October 2023, clonazepam was added for anxiety management. Upon confirmation of pregnancy in January 2024, clonazepam was discontinued given reproductive safety concerns. At the time of referral at 32 weeks' gestation, the patient was receiving brexpiprazole 1 mg daily and quetiapine 100 mg nightly, with no other psychotropic medications.

## 3 Differential Diagnosis, Investigations, and Treatment

The differential diagnosis included bipolar II disorder with hypomanic and depressive episodes, generalized anxiety disorder, and comorbid anxiety symptoms associated with pregnancy. There was no clinical evidence of psychotic disorder or substance‐induced mood disorder.

The risks and benefits of continuing brexpiprazole were discussed in detail, including the limited reproductive safety data, the off‐label nature of its use for bipolar disorder, and the high risk of relapse associated with treatment discontinuation. The FDA label notes that neonates exposed to antipsychotic drugs, including brexpiprazole, during the third trimester of pregnancy are at risk for extrapyramidal and/or withdrawal symptoms; this risk was discussed with the patient and monitoring was planned accordingly [2]. The patient elected to continue brexpiprazole and quetiapine.

During late pregnancy, quetiapine was titrated to 200–300 mg nightly to address anxiety, insomnia, and relapse prevention. A comprehensive postpartum care plan was developed emphasizing protective sleep, relapse monitoring, breastfeeding considerations, and trauma‐informed obstetric care.

## 4 Conclusion and Results (Outcome and Follow‐Up)

The patient delivered a healthy male infant via vacuum‐assisted vaginal delivery without obstetric complications. The infant weighed 3430 g at birth. Apgar scores were 8 at 1 min and 9 at 5 and 10 min. Neonatal adaptation was normal, with no extrapyramidal or withdrawal symptoms observed. During the first postpartum week, the patient experienced transient anxiety and mood lability that improved within 2 weeks with psychosocial support. Quetiapine was subsequently reduced to 150 mg nightly due to mild anticholinergic effects. Brexpiprazole 1 mg daily was continued, and the patient exclusively breastfed.

The infant was closely monitored through 6 months of age. Growth parameters, physical health, and neurodevelopmental milestones were age appropriate, with no observed adverse effects attributable to medication exposure. The mother remained psychiatrically stable without recurrence of mood episodes.

## 5 Discussion

Management of bipolar disorder during pregnancy requires balancing maternal psychiatric stability against potential fetal and neonatal risks. Discontinuation of mood‐stabilizing treatment is associated with high relapse rates, particularly in bipolar II disorder [10]. The prevalence of second‐generation antipsychotic use during pregnancy has increased from 0.4% to 1.3% over a decade [12].

Quetiapine is the most commonly prescribed atypical antipsychotic in pregnancy and has not been associated with an increased risk of major congenital malformations [12, 13]. Continuation of atypical antipsychotics may increase the risk of gestational diabetes, necessitating careful metabolic monitoring [14]. Regarding quetiapine use during lactation, the FDA label for quetiapine notes that the drug is present in breast milk; however, available data suggest that infant exposure is low, and most guidelines consider quetiapine compatible with breastfeeding with appropriate monitoring [15].

Evidence regarding brexpiprazole exposure during pregnancy and lactation remains extremely limited. Animal studies have not demonstrated teratogenicity [2]. As detailed in the Introduction, Konishi et al. [11] provided the first pharmacokinetic characterization of brexpiprazole in the perinatal period, demonstrating low but detectable placental transfer and low infant exposure via breast milk. It is important to note that brexpiprazole is not FDA‐approved for bipolar disorder, and its use in this case was off‐label [2]. Clinicians should communicate this to patients when considering brexpiprazole for perinatal management of bipolar disorder.

One clinically relevant consideration is the potential impact of brexpiprazole on breast milk production. A case report by Berlin and Bodnar [16] described low breast milk production in a lactating woman receiving brexpiprazole, raising the possibility of dopamine‐mediated suppression of prolactin and galactogenesis. In the present case, the patient was able to breastfeed exclusively without reported difficulties with milk supply, though this was not formally quantified. Clinicians should inquire about milk production in brexpiprazole‐exposed breastfeeding women.

This case should be interpreted with caution. The patient received polypharmacy (brexpiprazole and quetiapine), and quetiapine dosing was adjusted in late pregnancy and the early postpartum period. It is therefore not possible to attribute clinical stability to brexpiprazole alone; quetiapine dose adjustments may have been equally or more important contributors. This represents a significant limitation of the present report.

Additional limitations include the lack of standardized rating scales for mood symptoms (such as the Edinburgh Postnatal Depression Scale [EPDS], Patient Health Questionnaire‐9 [PHQ‐9], or Young Mania Rating Scale [YMRS])—mood status was assessed clinically through psychiatric interviews by a psychiatrist—and the absence of formal quantification of breast milk drug concentrations. Apgar scores (8 at 1 min, 9 at 5 and 10 min) are reported; however, comprehensive neonatal neurodevelopmental follow‐up beyond 6 months was not conducted, and cumulative effects of medication exposure cannot be excluded.

## Author Contributions

**Awateif Alsupiany:** conceptualization, data curation, formal analysis, funding acquisition, investigation, methodology, project administration, writing – original draft, writing – review and editing.

## Funding

The author has nothing to report.

## Consent

Written informed consent for publication was obtained from the patient and from the patient as the legal guardian for her infant.

## Conflicts of Interest

The author declares no conflicts of interest.

## Data Availability

The data supporting this case report are not publicly available in order to protect patient confidentiality; however, the data are available from the corresponding author upon reasonable request.
